# Pressure Induced Molecular‐Arrangement and Charge‐Density Perturbance in Doped Polymer for Intelligent Motion and Vocal Recognitions

**DOI:** 10.1002/adma.202500077

**Published:** 2025-04-08

**Authors:** Huimin Lu, Lei Zhang, Jingyan Jiang, Jian Song, Zhongchao Zhou, Wujian Wu, Ziqian Cheng, Tengfei Yan, Hong Hu, Tingting Zhao, Zhen Xu, Siyi Luo, Hui Li, Jianhua Zhang, Charles H. Lawrie

**Affiliations:** ^1^ School of Microelectronics Shanghai University Shanghai 201800 China; ^2^ Sino‐Swiss Institute of Advanced Technology (SSIAT) Shanghai University Shanghai 201899 China; ^3^ College of Big data and Internet Shenzhen Technology University Shenzhen 518118 China; ^4^ Graduate School of China Academy of Engineering Physics Beijing 100193 China; ^5^ State Key Laboratory of High Performance Ceramics and Superfine Microstructures Shanghai Institute of Ceramics Chinese Academy of Sciences Shanghai 200050 China; ^6^ Biogipuzkoa Health Research Institute San Sebastian 20014 Spain; ^7^ IKERBASQUE Basque Foundation for Science Bilbao 48009 Spain; ^8^ Radcliffe Department of Medicine University of Oxford Oxford OX3 9DU UK

**Keywords:** doped polymer, Pressure sensor

## Abstract

Conjugated polymers (CPs) show great potential for pressure detection due to the amorphous polymer packing, but a lack of clarity regarding sensing mechanisms hampers the development of further applications. Herein, a sacrificial template‐full solution method with both rough surface and high conductivity is described that can be applied to sandwich‐structured resistive pressure sensors. Transient absorption measurements demonstrate the significant increase of carrier lifetime (from 1.44 to 2.54 ns) induced by pressure, which directly evidenced the superior sensing mechanism of sidechain doped conjugated polymer. This sensor displayed low‐pressure detection limit of 0.7 Pa as well as a rapid response time of 18.8 ms, enabling multi‐mode motion analysis including wrist pulse, swallowing, finger bending, grabbing, and typing. Additionally, an intelligent vocal recognition system with convolutional neural networks is used which can achieve >96% classification accuracy across diverse vocal profiles. This general approach is anticipated and enables a new direction for the development of pressure sensors.

## Graphical abstract

The sidechain doped polymer is investigated to have great potential for pressure detection due to the advantages including molecular rearrangement and charge‐density perturbance induced by micro‐mechanical deformation. This sensor displayed low‐pressure detection limit of 0.7 Pa as well as a rapid response time of 18.8 ms, enabling multi‐mode motion analysis and intelligent vocal recognitions.

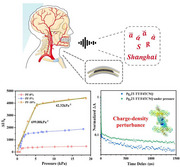

## 1 Introduction

Flexible pressure sensors have generated significant attention in recent years due to their ability to detect mechanical stimuli, making them eminently suitable for use in electronic skins,^[^ ^1^, ^2^, ^3^ ^]^ wearable devices^[^ ^4^, ^5^, ^6^ ^]^ and human‐computer interactions.^[^ ^7^, ^8^, ^9^ ^]^ At present, capacitive and resistive pressure sensors represent the two most extensively studied and widely focused types of pressure sensing technologies. The capacitance type pressure sensor could exhibit a relative high sensitivity, but these sensors have a high requirement for packaging to shield the electromagnetic interference around them, which could be a drawback for making wearable devices. Hence, the development of piezoresistive sensors become important for the advantages including low cost and small volume.^[^ ^10^, ^11^, ^12^ ^]^ Organic semiconductors are a promising choice for piezoresistive sensors owing to their natural flexibility, which can convert pressure stimuli into a change in resistance.^[^ ^13^, ^14^, ^15^, ^16^, ^17^ ^]^ Typically, this device comprises of two electrodes and a sensing layer sandwiched between them.^[^ ^18^, ^19^ ^]^ Although flexible pressure sensors based on organic semiconductors offer advantages such as high drift stability and a simple structure, their sensitivities are limited, and their resolutions are maintained within a narrow pressure range. Engineering the organic semiconductor thin film with microstructured surfaces has proven to be an effective method of improving both sensitivity and response speed.^[^ ^15^, ^20^, ^21^, ^22^, ^23^, ^24^, ^25^, ^26^ ^]^ These microstructured surfaces can increase the contact area between the electrode and sensing layer during deformation induced by mechanical stimuli. However, due to the lack of inside resistance change, the sensing performance of these sensors are still not responsive to micro pressure changes.^[^ ^27^, ^28^ ^]^ Consequently, there has been emerging interest in deformation‐sensitive organic semiconductor materials to further enhance sensing performance.

Conjugated polymers (CPs) are a common type of organic semiconductor material. Unlike the crystalline structures of most inorganic materials, conjugated polymer thin films are formed by π‐π stacking, resulting in a much lower Young's modulus,^[^ ^29^, ^30^ ^]^ making them exceptionally promising candidates for flexible pressure sensor development. The movement of carriers along π‐π stacking directions is influenced by the molecular arrangement due to the π‐conjugated structure between molecular chains, making the pathway deformation sensitive. However, measuring the resistance of conventional CPs directly is not feasible due to its high resistance, which results in low signal to noise ratios. Chemical doping can be used to increase the conductivity of conjugated polymers,^[^ ^31^, ^32^, ^33^ ^]^ and dopants with suitable energy levels can introduce large numbers of carriers into the polymer. For example, Su et al^[^ ^24^ ^]^ produced a conductive polymer (poly(3,4‐ethylenedioxythiophene):poly(styrenesulfonate), PEDOT:PSS) and a soft polymer (poly(2‐acrylamido‐2‐methyl‐1‐propanesulfonic acid), PAAMPSA) composite film that was utilized as a tactile sensor with a pressure sensitivity of 164.5 kPa^−1^ and response time of 19 ms. However, in this case the dopant used was chemically connected to the backbone of PEDOT restricting sensitivity to mechanical deformation. An alternative solution involves inserting the dopant on the sidechain of the CP and fixing it through donor‐acceptor interaction.^[^ ^34^ ^]^ In this case the distance between the dopant and polymer backbone can directly influence the pathway of the carrier resulting in a change to the carrier injection status allowing for further improvement to the sensing performance of flexible pressure sensors based on CP technology.

Below we propose a sacrificial template‐full solution (ST‐FS) method for fabricating blended thin films comprising of CPs, dopants, and sacrificial templates. This method allows for a combination of high conductivity and a rough surface on the CP layer, both of which are key factors in the development of sensitive pressure sensors. More importantly, we revealed the sensing mechanism in molecular level of this sidechain doped polymer thin film, which could bring more significant resistance change inside it and further enhance the sensing performance. The results of transient absorption measurement toward different polymer thin films with and without pressure also offered an important insight into the sensing mechanism. The carrier lifetime could be easily influenced by pressure on the sidechain doped polymer, but this phenomenon could be hardly observed on pristine polymer without dopant. According to the results of density functional theory analysis, we indicated that the underlying causes could be the molecular rearrangement and charge‐density perturbance induced by pressure inside the polymer thin film. We demonstrated the validity of the carrier mobility‐lifetime relationship by using chemical doped polymer thin film, which is already widely accepted in inorganic semiconductors. The fabricated sensor exhibited a high sensitivity of 699.8 kPa^−1^ at a range of 0–5.4 kPa, a fast response time of 19 ms and a low‐pressure detection limit of 0.7 Pa. Furthermore, the sensor was found to be suitable for multi‐mode motion analysis including wrist pulse, swallowing, finger bending, grabbing, and typing sensor activities. We further demonstrated the integration of an advanced speech recognition system based on convolutional neural networks with a classification accuracy of over 96% across diverse vocal profiles. Compared with the previous research, the fast response speed of this sensor could enable a more detailed sensing signal record toward vocal vibration, which provided more features for convolutional neural networks assisted vocal recognition.^[^ ^12^ ^]^ In summary, this study presents a simple fabrication method for CP‐based thin films and demonstrates the sensing mechanism and versatile applications of the developed pressure sensor.

## 2 Results and Discussion

### 2.1 Materials and Sensing Mechanism

**Figure 1:**
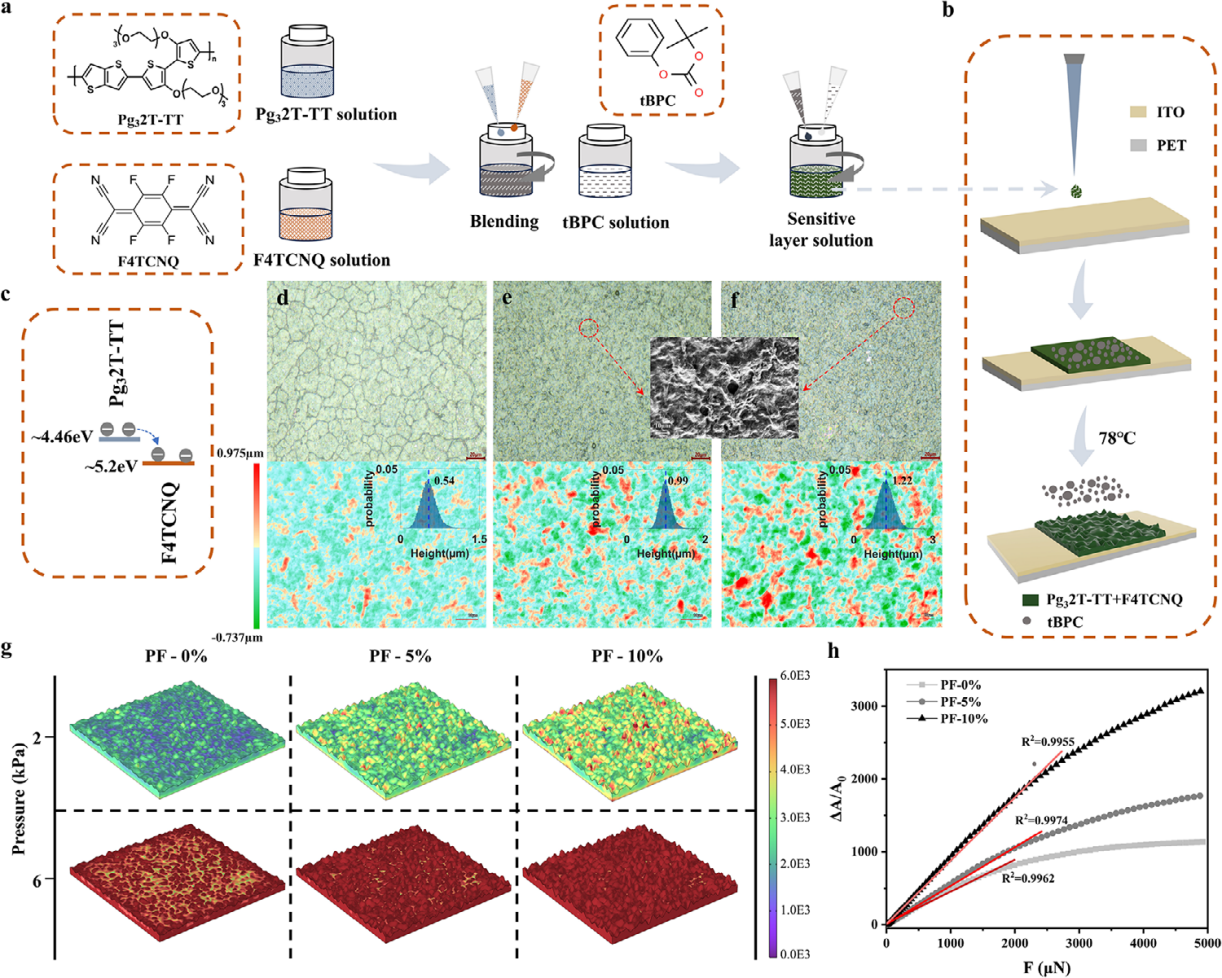
a) Chemical structures of Pg_3_2T‐TT, F4TCNQ, and tBPC. b) Sensor layer preparation. c) Doping mechanism of Pg_3_2T‐TT/ F4TCNQ. d–f) Optical image (top) and 2D height map (bottom) of PF‐0%, PF‐5% and PF‐10%, respectively. g) Finite element analysis results of PF‐0%, PF‐5%, and PF‐10% contact surfaces at loading pressures of 2 and 6 kPa, respectively. h) Variation of contact area with applied pressure for GRR model with different roughness in FEA model.

Changes in the resistance of piezoresistive pressure sensors occur at the macro level, caused by the sensing layer and electrode and at the microscopic level, caused by resistance changes within the sensing layer itself.^[^ ^35^ ^]^ The value of the interfacial contact resistance between the electrode layer and the sensing layer decreases when the sensor is under pressure. Sensors with microstructures can increase the effective contact area between the sensor surface and the electrode layer and then improve the sensitivity.^[^ ^36^ ^]^ We used the ST‐FS to achieve a uniformly doped CP thin film with randomly distributed rough surfaces. 2,3,5,6‐tetrafluoro‐tetracyanoquinodimethane (F4TCNQ) was added to the Pg_3_2T‐TT solution as the dopant and mixed well as shown in **Figure** **1**a. Due to the matching of F4TCNQ and Pg_3_2T‐TT energy levels, the electrons on the HOMO energy level in the conjugated polymer are transferred to the LUMO energy level in F4TCNQ (Figure 1c), which achieves an increased charge density and improves the conductivity of this thin film. Tert‐butyl phenyl carbonate (tBPC) solution was then added to the Pg32T‐TT/F4TCNQ solution and mixed, whereas the responsive layer of the pressure sensor was prepared using the drop‐casting method, with tBPC as the sacrificial template. As shown in Figure 1b, the mixed Pg_3_2T‐TT/F4TCNQ/tBPC solution was drop‐casted on the ITO/PET layer and heated to 78 °C. Due to the low boiling point of tBPC (≈ 78 °C), it was removed by evaporation leading to the production of irregular microstructures containing holes on the doped CP thin film. The volatilization of tBPC leaves hollows and spine‐like structures randomly on the surface, which was proved to be a key factor to achieve good sensing performance.^[^ ^23^ ^]^ We produced Pg_3_2T‐TT/F4TCNQ thin films in the absence of tBPC (PF‐0%) as well as with 5% (PF‐5%) and 10% (PF‐10%) tBPC in weight as shown in Figure 1d– f (top), respectively. The corresponding Pore size distribution diagram and film cross‐section SEM images are shown in Figure S1 (Supporting Information). According to the result, the total volume of holes significantly increased under the effect of tBPC. The size of these porous structure is around 5–20 nm, indicating a uniform distribution of tBPC inside the polymer thin film. Besides, the cross‐sectional images of PF‐0%, PF‐5% and PF‐10% were provided by using scanning electron microscope. The layered structure also became obvious after tBPC was used. However, the sensing layer became discontinuous when too much tBPC (PF‐15%) was applied (Figure S2, Supporting Information). The surface microstructures of the films were also investigated by using laser confocal (Figure S3, Supporting Information), which all conform to the Gaussian‐distributed random roughness microstructure (GRR).

Figure 1d–f (bottom) illustrates the 2D height distribution of PF‐0%, PF‐5% and PF‐10%, as well as the 3D morphology (Figure S4, Supporting Information). In order to further explore the influence of surface microstructures, we simulated the compressive deformation behavior of the GRR using finite element analysis (FEA). Based on the surface profile parameters of the sensing layer measured by laser confocal, a model of the PF sensing layer of the GRR structure was developed, and the model meshed using densely arranged free triangles. The Figure 1g shows the stress distribution of the PF‐0%, PF‐5%, and PF‐10% models at 2 and 6 kPa applied force. It can be observed that the internal stresses are first concentrated at the highest peak point of contact. As the pressure increases, the stress distribution shifts to peaks and valleys or other low protruding peak points. The contact peaks of PF‐0%, PF‐5% and PF‐10% increased sequentially at 2 kPa, corresponding with the increase of the conductive paths of the three sensors in turn. At 6 kPa, the upper and lower conductive paths of all three sensors were almost completely connected indicating a gradual saturation of the electrical signal. The sensitivity at this stage is much lower than at the first stage, although the sensitivities remained different. According to the equivalent resistance diagram of the sensor (Figure S5, Supporting Information), Re is the electrode resistance and Rs is the contact resistance, since Re is much smaller than Rs, the relative current change of the sensor is expressed as follows: $\frac{\Delta I}{I_{0}}=\frac{R_{0}}{R_{s}}\;-1$, and the contact resistance:$R=\frac{\rho }{A}$, where *ρ* is the resistivity of the sensing material and *A* is the contact area, and then the sensitivity $S=\frac{\Delta I/I_{0}}{\Delta P}$, thus $\frac{\Delta I}{I_{0}}\propto \frac{\Delta A}{A_{0}}$, where A_0_ is the contact area before pressure is applied and ΔA is the change in contact area A. Figure 1h shows the rate of change of contact area (ΔA/A_0_) as a function of load. It can be observed that PF‐0% demonstrated the smallest linear response range, while PF‐10% has the largest linear response range probably due to the rough and wide surface height distribution interval. When the higher peaks gradually decrease and flatten out with increasing load, the lower sharp peaks compensate for the decrease in sensitivity due to flattening, thus extending the linear response range of the sensor.^[^ ^23^ ^]^

**Figure 2:**
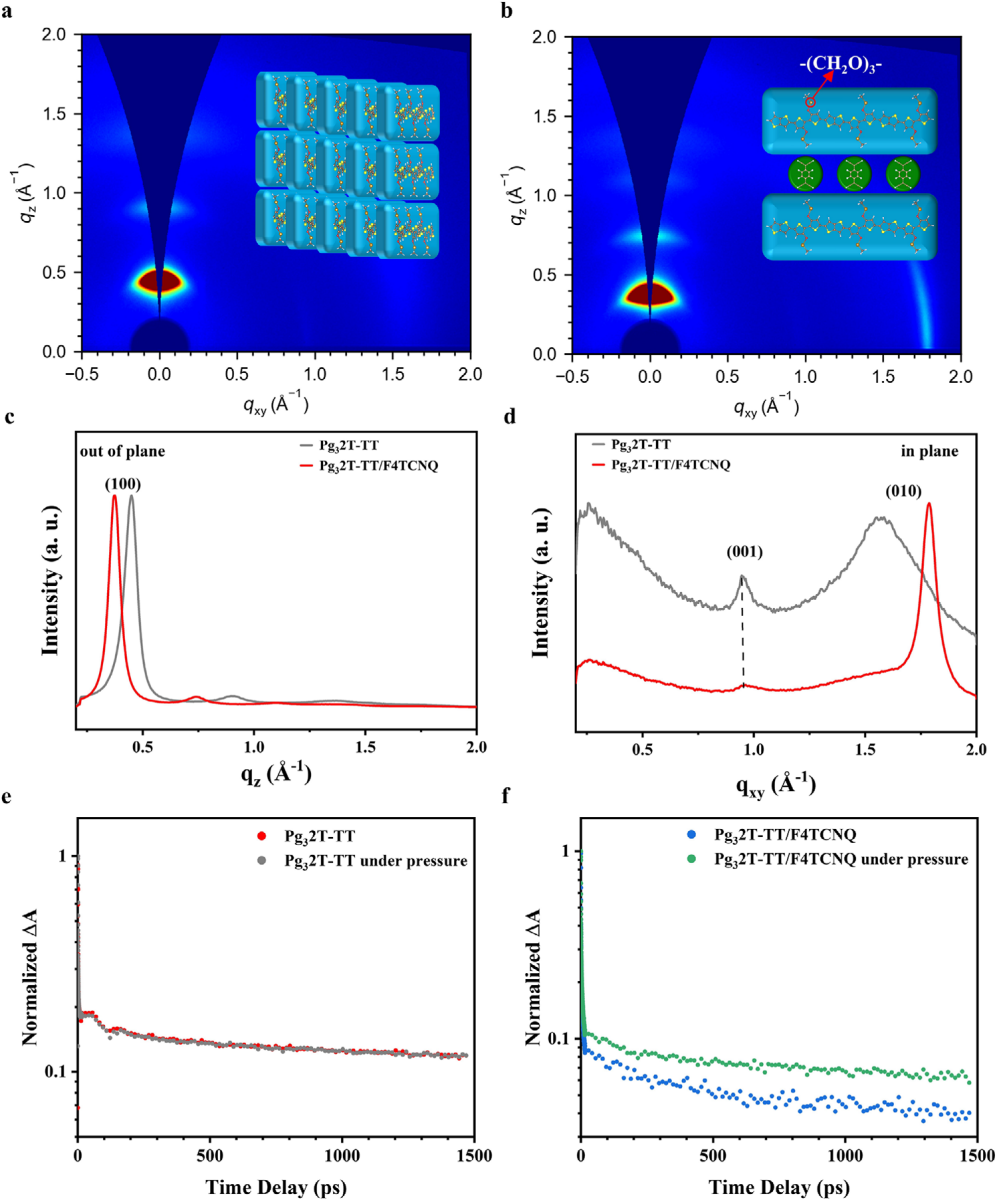
The GIWAXS results of a) pure Pg_3_2T‐TT and b) 10% wt F4TCNQ doped Pg_3_2T‐TT. c) Out of plane and d) In plane line‐cut profiles with and without dopant. The decay curves of TA for e) Pg_3_2T‐TT and f) Pg_3_2T‐TT/F4TCNQ with and without pressure.

Grazing incidence wide‐angle X‐ray scattering (GIWAXS) measurements were performed to measure the polymer packing upon doping. As can be seen from **Figure** **2**a, pristine Pg_3_2T‐TT shows an edge‐on orientation due to the clear (*h*00) peaks along q_z_ direction and (010) peak along q_xy_ direction. The edge‐on orientation was maintained upon doping (Figure 2b). Compared with pristine film, the lamellar stacking distance of doped film increases from 13.96 to 16.75 Å (Figure 2c) and π‐ π stacking distance decreases from 4.0 to 3.51 Å (Figure 2d) after doping by 10% wt F4TCNQ, indicating the dopant inserting between side chains without interrupting the packing of polymer backbone.

Transient absorption (TA) measurements were applied on Pg_3_2T‐TT and Pg_3_2T‐TT/F4TCNQ using a home‐built setup for pressure adjustment. The decay curves of TA line‐cut within the ground state bleaching region are shown to compare the carrier lifetime (Figure 2e,f). The data was averaged around a 50 nm window. The lifetime of the Pg_3_2T‐TT/F4TCNQ under pressure is longer compared to its freeform condition (Figure 2f), while the Pg_3_2T‐TT shows no observable lifetime difference with or without pressure (Figure 2e). The carrier lifetime of Pg_3_2T‐TT and Pg_3_2T‐TT/F4TCNQ thin film were deduced by exponential decay fitting to be 2.54 ± 0.05 ns and 1.44 ± 0.05 ns without pressure. The carrier lifetime of Pg_3_2T‐TT/F4TCNQ increased to 2.04 ± 0.11 ns under pressure while that of Pg_3_2T‐TT rarely changed. Combining with the GIWAXS results, the lifetime difference could be concluded as follow: the lamellar stacking distance show strong relationship with the carrier lifetime, and the distance of Pg_3_2T‐TT could be hardly influenced by pressure due to the molecular steric hindrance. But the extra distance brought by dopant make it possible to have a molecular level rearrangement on lamellar stacking under pressure thereby reduce the lamellar stacking distance. According to the previous research, the carrier lifetime and mobility are linearly depended^[^ ^37^ ^]^: $\mu =\tau_{sc}\frac{q}{m_{n}}$, where μ is the carrier mobility, τ_*sc*_ is the carrier lifetime, *m_n_* is effective mass and q is the electric charge. Hence, the pressure induced carrier lifetime extension could directly enable a higher carrier mobility, which could further enhance the perpendicular direction conductivity.

We demonstrated the validity of the carrier mobility‐lifetime relationship by using chemical doped polymer thin film, which is already widely accepted in inorganic semiconductors. And the advantage of sidechain doped polymer‐based pressure sensor could be summarized as follow: The formation of pristine polymer thin film is mainly induced by π‐π stacking, and the carrier lifetime could be hardly influenced by pressure. The sensitivity of pristine polymer‐based pressure sensor is only attributed by the changing contact area between polymer and electrode, but the conductivity of the polymer thin film itself rarely changed. After the sidechain of this polymer was doped, the carrier lifetime could be directly influenced by pressure, which means the sensitivity was synergistically attributed by both the changing contact area and the conductivity change inside this thin film. Hence, the sensitivity of sidechain doped polymer is much higher than the pristine one.

**Figure 3:**
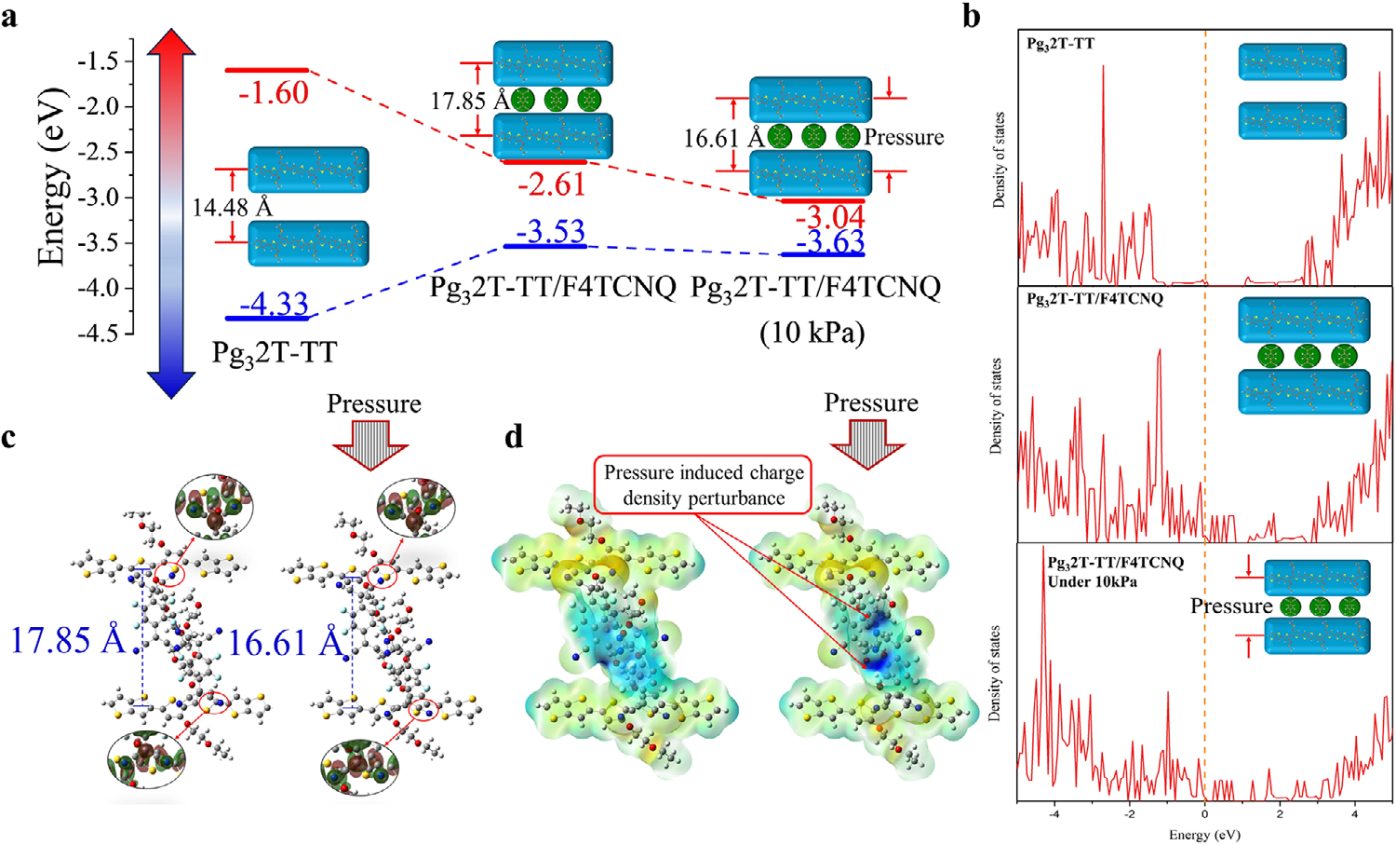
a) The calculated bandgap width of Pg_3_2T‐TT, Pg_3_2T‐TT/F4TCNQ before and under pressure. b) The total DOS of Pg_3_2T‐TT, Pg_3_2T‐TT/F4TCNQ before and under pressure. c) Orbital overlap between Pg_3_2T‐TT and F4TCNQ before and under pressure. d) Electrostatic potentials of Pg_3_2T‐TT and F4TCNQ before and under pressure.

DFT analysis was then applied to further investigate the interaction between Pg_3_2T‐TT and F4TCNQ molecules under pressure conditions. The corresponding molecular structures were constructed according to our GIWAXS results. Structural optimization of all species was carried out by the VASP software package given in Figure 3b. The pristine Pg_3_2T‐TT exhibits semiconducting properties as can be seen from **Figure** **3**a,b. After being doped by F4TCNQ, the outer orbital electrons of Pg_3_2T‐TT and F4TCNQ were observed to interact resulting in changed the electronic structure of the system. The Fermi energy level moved ≈ 1.30 eV toward the low energy state and entered the valence band, so that a hole state is formed at the top of the valence band. When 10 kPa pressure was then applied to the doped system, the calculated lamellar stacking distance decreased from 17.85 to 16.61 Å, while the bandgap decreased to 0.59 eV. According to the Fermi‐Dirac distribution function ($n=\frac{1}{e^{(\varepsilon -\mu )/kT}+1}$ (n is the particle occupation probability, *ε* is the energy, μ is the chemical potential which is the Fermi energy level, and T is the temperature)), the position of the Fermi energy level affects the concentration of electrons and holes in the material, which in turn affects the conductivity. Chemical doping could increase the electron concentration of Pg_3_2T‐TT, so that the position of the Fermi energy level decreases leading to an increase in conductivity. The molecular orbital overlap and the Electrostatic potentials (ESP) of the different systems were calculated using the Gaussian 16 software package as shown in Figure 3c,d. It can be observed that the area of orbital overlap between F4TCNQ and Pg_3_2T‐TT becomes larger under pressure (Figure 3c), and doping position had a positive electrostatic potential (Figure 3d) with the electrostatic potential becoming greater under pressure suggesting an enhanced interaction between F4TCNQ and Pg_3_2T‐TT associated with a decrease in the bandgap of the system. In other words, as the bandgap becomes smaller after doping, and the Fermi energy level enters the valence band, this improves the conductivity of Pg_3_2T‐TT. As the interaction between F4TCNQ and Pg_3_2T‐TT is enhanced, and the bandgap is reduced, leading to pressure induced charge density perturbance, there is a further enhanced sensitivity toward micro mechanical deformation.

### 2.2 Sensing Performance and Applications

**Figure 4:**
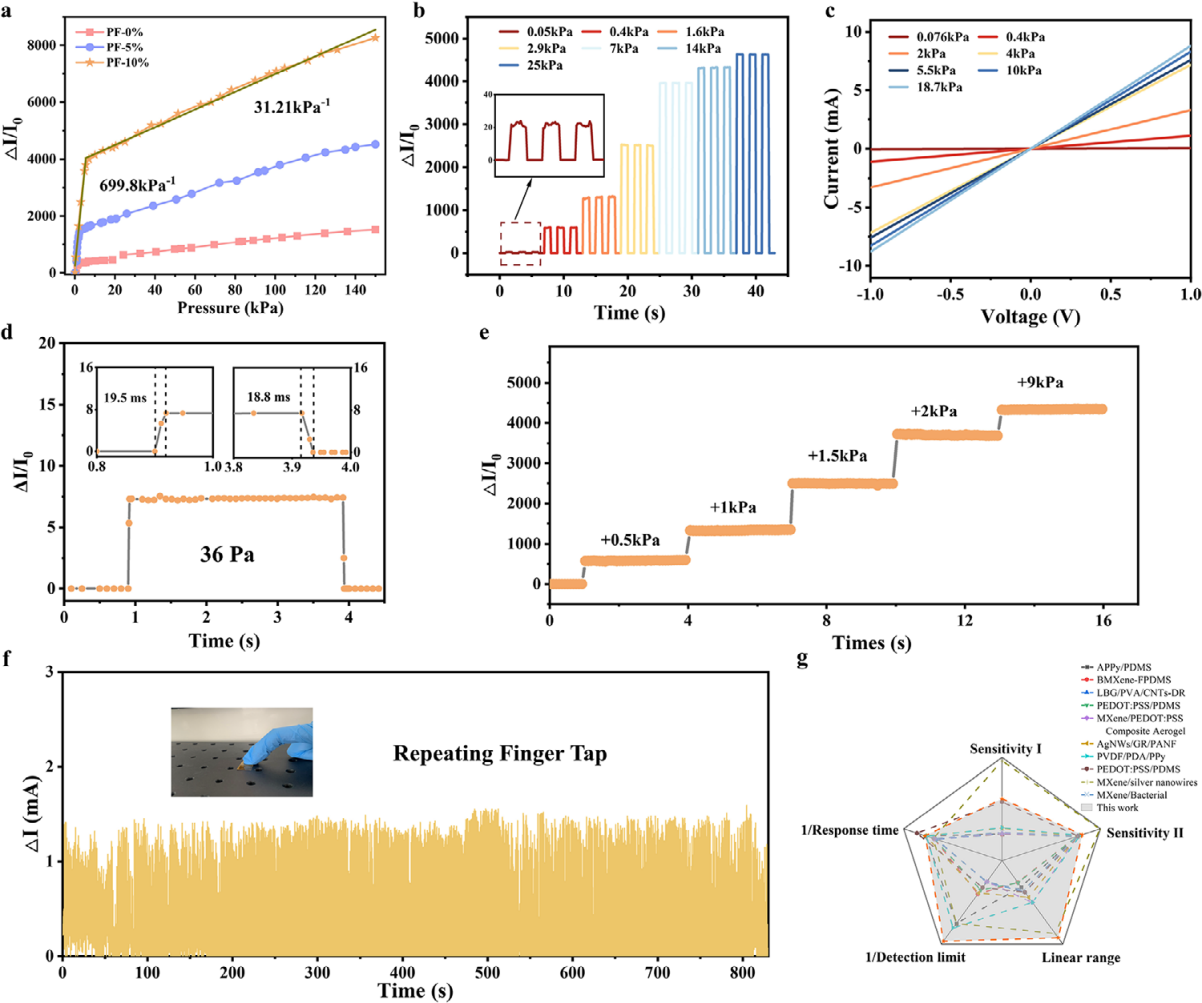
a) Sensitivity of three GRR pressure sensors. b) ΔI/I_0_‐T curves of the PF‐10% sensor under serial pressures. c) *I–V* curves of the PF‐10% sensor under serial pressures. d) response and recovery time of the sensor. e) Dynamic response of the PF‐10% sensor under a step pressure loading. f) Finger taping during 800 s. g) Performance comparison of our pressure sensors with existing piezoresistive pressure sensors.

In evaluating the performance of a sensor, it is essential to consider key parameters such as sensitivity, detection range, response time, and stability.^[^ ^38^ ^]^ Sensitivity (S) is defined as the rate of change of the electrical signal output from a pressure transducer per unit of pressure. It is typically calculated using the slope of the device's “rate of change of electrical signal (ΔI/I_0_) – pressure (ΔP)” curve. This can be expressed as S = (ΔI/I_0_)/ ΔP in kPa^−1^, where I_0_ is the initial current, ΔI is the relative change in current, and P is the pressure corresponding to the applied pressure.^[^ ^39^ ^]^ To evaluate the influence of dopant concentration, PF‐0% and PF‐10% were prepared with varying doping ratios (Figure S6, Supporting Information). The rate of current change increased with the doping ratio and became saturated at 0.2 for both PF‐0% and PF‐10%, further indicating the importance of sidechain doping. Notably, the response value of both PF‐0% and PF‐10% decreased at the doping ratio of 0.25, which mainly attributed to the increasing base line current (I_0_). From these results shown in Figure S6c,d (Supporting Information), it is evident that the PF‐10% device exhibits a much lower baseline current (I₀) compared to the PF‐0% device. The lower baseline current (I₀) in the PF‐10% device can be attributed to the increased surface roughness induced by tPBC during fabrication. The rougher surface reduces the effective contact area between the sandwich‐structured pressure‐sensitive layer and the electrode. This reduction in contact area decreases the baseline conductivity of the sensor, resulting in a lower I₀. Additionally, the reduced I₀ in the PF‐10% device enhances its relative current change (ΔI/I₀) under pressure, thereby improving the sensitivity of the pressure sensor. In contrast, the smoother surface of the PF‐0% device increases the contact area with the electrode, leading to a higher baseline current (I₀) and consequently reducing the relative current change and sensitivity. These data provide further insight into how surface roughness directly influences the electrical and mechanical performance of the pressure sensor. These results indicated that the doping ratio need to be kept in an appropriate amount to ensure good sensing performance. Hence, the doping ratio of 0.2 was applied in the following experiments because of the best sensing performance (Figure S7, Supporting Information). The encapsulated pressure sensor and the size was shown in Figure S8 (Supporting Information). The current change‐pressure curve can be divided into two parts according to the sensitivity, in the first stage, the corresponding sensitivity is S_1_, and in the second stage, the corresponding sensitivity is S_2_ (**Figure** **4**a). For PF‐0% (Figure S9a, Supporting Information), when the applied pressure is less than 1.6 kPa, the sensitivity S_1_ of the device was 424.66 kPa^−1^, wheras when greater than 1.6 kPa, the sensitivity S_2_ of the device was 7.8 kPa^−1^. For PF‐5% (Figure S9b, Supporting Information), the sensitivity S_1_ of the device was 593.55 kPa^−1^ when the applied pressure was less than 3 kPa, and when greater sensitivity S_2_ was 21.4 kPa^−1^ a. For PF‐10% (Figure S9c, Supporting Information), when the applied pressure was less than 5.4 kPa, sensitivity S_1_ of the device was 699.80 kPa^−1^ and at higher pressures 31.21 kPa^−1^. These results were consistent with those of the simulation.

Consequently, we selected PF‐10% for further performance testing. The current rate‐of‐change‐time (ΔI/I_0_‐T) curve (Figure 4b) showed a gradual increase in current with the gradual increase in external pressure. The current‐voltage (I‐V) curve of the pressure sensor over the dynamic voltage range (‐1.0 to 1.0 V) (Figure 4c) was linear and the slope increases with pressure, which is related to the formation of an ohmic contact between the polymer sensitive layer and the ITO electrode. The response performance of the sensor was tested at low (36 Pa) (Figure 4d) and high (150 kPa) (Figure S10, Supporting Information) pressures. At 36 Pa, the response time was 19.5 ms while the recovery time was 18.8 ms. Furthermore, the dynamic response and stability of the sensor were investigated (Figure 4e). A certain pressure was applied to the sensor sequentially and held for 3 s, and the results showed that each pressure increment led to a stepwise increase in the rate of change of the current, and the response was rapid, and the current signal was stable. When the applied pressure accumulates in the pressure range corresponding to S_1_ and S_2_ in Figure 4a, the change of the current signal increment with the pressure increment follows the same trend as that observed in Figure 4a. In order to evaluate the mechanical stability of the sensors, we performed cyclic bending tests on the sensors (Figure S11, Supporting Information). According to the results, the response signal of both PF‐0% and PF‐10% rarely changed after 3000 bending cycles, which indicated a good durability as a wearable device. Besides, we fabricated two identical sensor devices for comparison. One sample was used as a control, where its sensitivity and microstructure were directly tested and analyzed using SEM imaging. The second sample underwent 5000 cycles of repeated pressing at a pressure of 150 kPa before its sensitivity and microstructure were tested under the same conditions. According to the results shown in Figure S12 (Supporting Information), both the control sample and the cycled sample demonstrated consistent sensitivity, with no significant degradation observed after the 5000 high‐pressure cycles. The SEM images of the cycled sample showed no visible cracks, deformation, or structural degradation in comparison to the control sample. This indicates that the sensor material maintained its mechanical integrity even after prolonged exposure to high‐pressure conditions. The PF‐10% based sensor was also tested by continuous finger taping during 800 s with random pressure (Figure 4f), and the result could help us further illustrate the potential for repeated use. To evaluate the environmental stability of the sensor, our sensor was tested under different varying temperature (‐20 to 80 °C) and humidity (10% to 90% RH) conditions (Figure S13, Supporting Information). The sensing performance rarely changed during the change of both temperature and humidity, which might be attributed by the packaged structure by using PI film on both sides of this sensor. We re‐tested the sensor fabricated two months ago to assess changes in sensing performance (Figure S14a, Supporting Information). The results indicate that the sensitivity of the sensors remained consistent, with no significant degradation observed after two months of storage under ambient conditions. To further validate the stability, we fabricated a new sensor and performed weekly measurements of its sensitivity. The data shown in Figure S14b (Supporting Information) demonstrate that the sensors exhibit excellent stability, with less than 5% variation in sensitivity throughout the testing period. These results confirm that the applied package structure effectively protects the sensors from the impact of oxygen and humidity, ensuring long‐term stability. The performance (S_1_, S_2_, linear range, response time, detection limit and Linear range) of the PF‐10% sensor was compared with that of already developed flexible piezoresistive pressure sensors. As can be seen from Figure 4g and Table S1 (Supporting Information), the PF sensors not only exhibited high sensitivity and wide linear range performance at low pressures but were also displayed superior sensitivity over the high‐pressure range.

**Figure 5:**
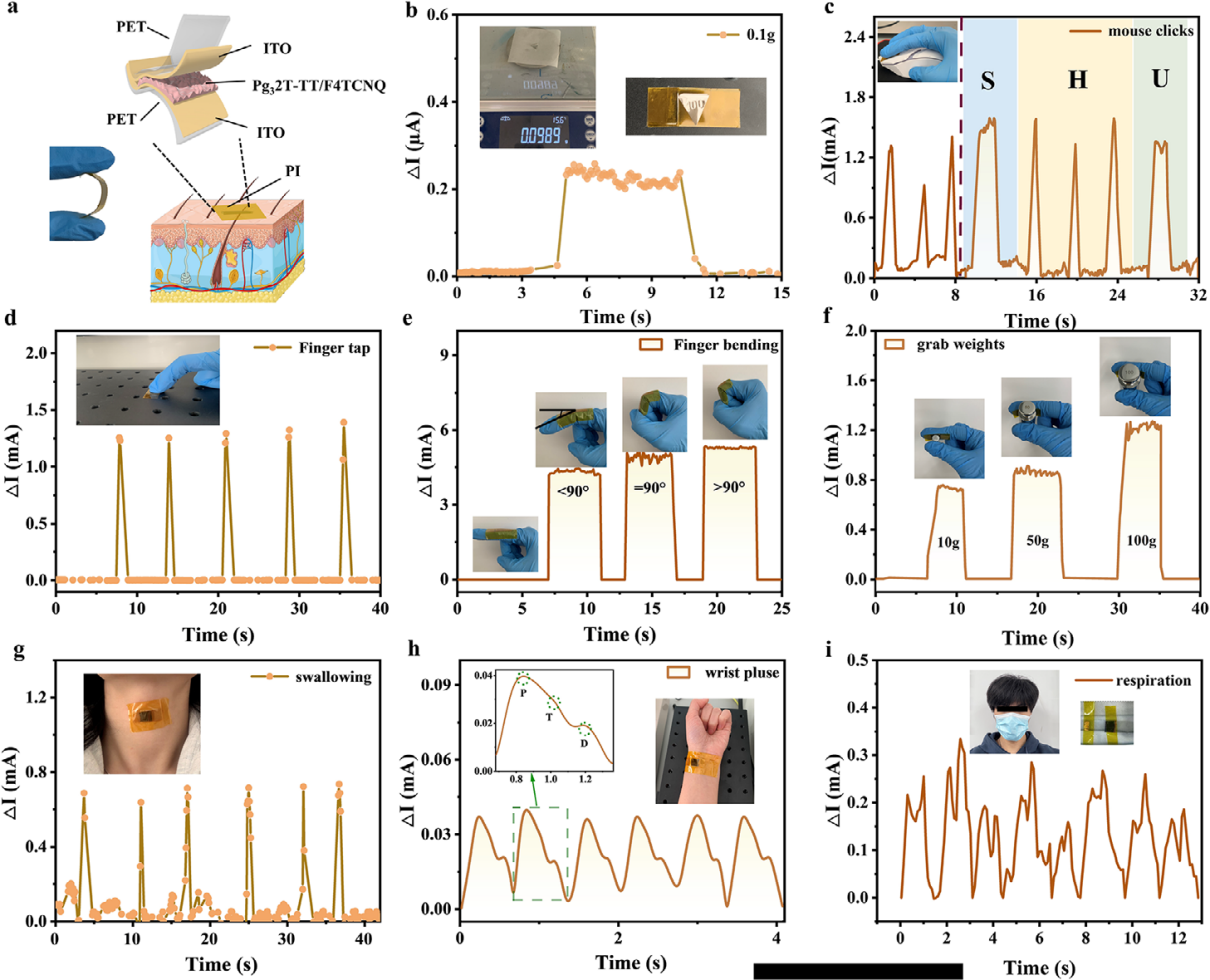
a) Schematic diagrams of Pg_3_2T‐TT/F4TCNQ based pressure sensor. Applications of the PF‐10% sensor in the detection of minute pressures and real‐time monitoring of human activities. b) Monitoring low hydrostatic pressure at 0.1 g tablet code with a PF‐10% sensor. c) Mouse clicks. d) Finger tap. e Finger bending. f) Grab weights. g) Swallowing. h) Wrist pulse. i) Respiration.

As illustrated in **Figure** **5**a, the sensor exhibits good flexibility, and when the sensor was encapsulated with PI tape it could easily be attached to curved surfaces on the human body for the real‐time monitoring of human activity. Figure 5b illustrates the capacity of the pressure sensor to monitor minute pressures such as a mass of ≈100 mg (equivalent to115 Pa). Based on the triple signal‐to‐noise ratio, the detection limit of this sensor was calculated to be 0.7 Pa. The sensor can also be fixed to curved (Figure 5c) and flat (Figure 5d) objects to monitor the force. This sensor was also attached on the mouse which was using for drawing on the computer. A sensing curve was obtained in real‐time with mouse click, indicating the ability to respond dynamic changes in pressure as same as the sensor inside the mouse. (Movie S1, Supporting Information).

Next, we tested real‐time monitoring of swallowing by affixing the sensor to the relevant areas of the human body (Figure 5g). As the pressure sensor is subjected to changes in pressure and tension when it is bent leading to deformation of the sensing layer the conductive path of the sensor also changes leading to detection with the change of the response current changing with the angle of bending of the finger (or other skin surface containing musculature) (Figure 5e), allowing subtle changes in human behavior to be monitored through the quantitative analysis of the response current. Furthermore, when we attached the flexible pressure sensor to the inner part of the finger, the output signal of the sensor changed when grasping different weights and holding them for a period of time (Figure 5f). In addition, by attaching the sensor tightly to the wrist artery, the pulse beat can be detected in real time (Figure 5h). Amplifying a single pulse wave, the pulse head (P‐wave), tidal (T‐wave), and diastolic (D‐wave) waves can be clearly obtained, which demonstrates the potential application of the sensor in vital signals detection. By integrating the sensor with the mask, the response curve based on each breathe could be clearly detected by this sensor (Figure 5i), which could enable the application in the monitoring system for acute respiratory distress syndrome.

### 2.3 Convolutional Neural Networks Assisted Vocal Recognition

**Figure 6:**
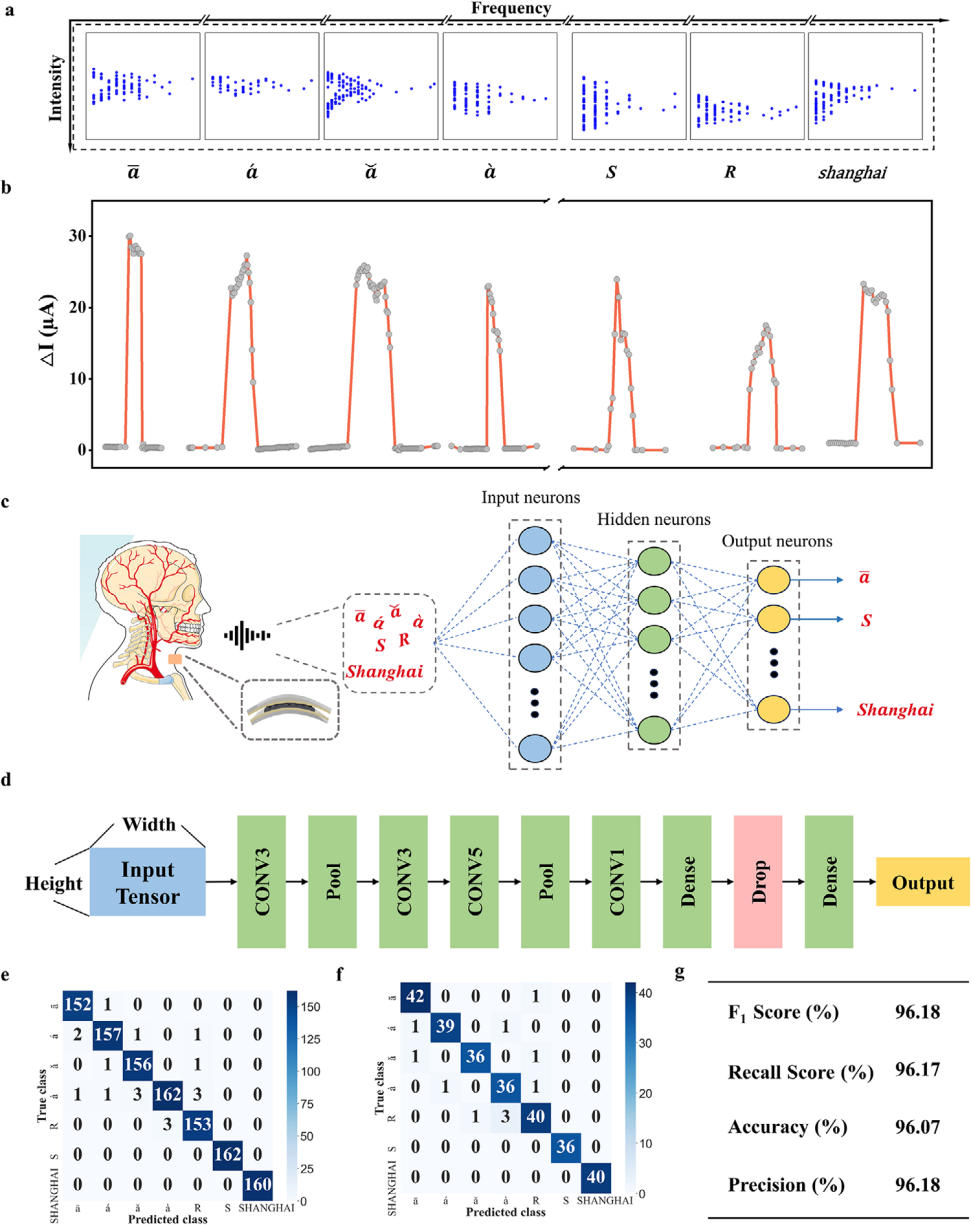
a) Frequency‐Intensity related data collected by this sensor. b) Current response curves for each group of sounds. c) Schematic flowchart of speech recognition. d) The architecture of classifier for acoustic data. e) Confusion matrix of the speech prediction versus the train data set. f) Confusion matrix of the speech prediction versus the test data set. g) Performance of 1D CNN models on multi‐category acoustic datasets.

Speaking is one of the most important ways of human communication and human‐computer interaction. Flexible pressure sensors can be used to capture the larynx vibrations produced by sound and convert the sound signal into a pressure distribution signal. The sensing signal will change due to the movement of the laryngeal muscles, thus collecting different sound signals and tones. Tones are of particular importance to the Chinese language, as they can change the pronunciation and meaning of words and are essential to the accurate understanding and expression. As can be seen from **Figure** **6**b, wearing the sensor and saying the representative letters “S” and “R” in the alphabet, the Chinese words flat tone ($\bar{a}$), up tone (*á*), go tone (*ǎ*), and enter tone (*à*), and the word “*shànghǎi*”, different current response curves were obtained, which indicated the ability to recognize different subtle laryngeal vibrations. In order to fully demonstrate the information of the data values, we repeated the acquisition of each sound group 20 times and then processed the data with Frequency‐Intensity related shown in Figure 6a. This data processing method could enable clear observations of various larynx vibrations, which show potential for deep learning assisted intelligent vocal recognition.

In order to develop sound recognition further we developed a compact 4‐layer 1D CNN for multi‐class classification of acoustic sensor data.^[^ ^40^ ^]^ The model takes 93‐D 1D vectors as input, representing acoustic measurements from different sensors. As illustrated as Figure 6d, the architecture consists of 4 convolutional layers (filter sizes 64, 128, 256, 512; kernel sizes 3, 3, 5, 1) to extract spatial features, each followed by ReLU activation.^[^ ^41^ ^]^ Max pooling layers (kernel size 2, stride 2) after the first 3 convolutional layers reduce dimensions. A dropout layer (p = 0.3) mitigates overfitting. The convolutional outputs are flattened and fed into 2 fully connected layers (FC1: 1024 units, FC2: 7 nodes for output classes). We trained the model on a dataset of 1400 samples labeled into 7 categories (ā, á, etc), with 200 samples per class. Preprocessing ensured data reliability for training. Figure. 6e,f presents the confusion matrices for the classification results on the training and testing datasets, respectively. The model demonstrates high accuracy across all classes in the training set (Figure 6e), with minimal misclassifications. For instance, classes “$\bar{a}$”, “***á***”, “***ǎ***”, “***à***”, “*R*”, “*S*” and “*sh* ***à*** *ngh* ***ǎ*** *i*” have correct predictions of 158/160, 157/160, 158/160, 158/160, 155/160, 156/160 and 160/160, respectively. Similarly, in the testing set (Figure 6f), the model maintains high accuracy, with classes “$\bar{a}$”, “***á***”, “***ǎ***”, “***à***”, “*R*”, “*S*” and “*sh* ***à*** *ngh* ***ǎ*** *i*” achieving correct predictions of 38/40, 39/40, 39/40, 36/40, 37/40, 40/40, and 40/40, respectively. These results underscore the model's robustness and effectiveness in accurately classifying training and testing datasets.

Our proposed 1D CNN model achieved excellent classification performance on the multi‐category acoustic dataset, as depicted in Figure 6g. Using the Adam optimization algorithm coupled with cross‐entropy loss, represented by:

(1) $$Loss=-\sum_{i\;=1}^{N}y_{i}\log\left({\hat{y}}_{i}\right)$$

where ***N*** is the number of samples, ***y***_***i***_ is the true label for sample of ***i***, ${\hat{y}}_{i}$ is the predicted probability for sample ***i***. The model achieved an F_1_ score of 96.18%, recall of 96.17%, accuracy of 96.07%, and precision of 96.18% on the test set. These metrics are defined as follows:

(2) $$\mathrm{Accuracy}\;=\frac{1}{N}\;\sum_{i=1}^{N}1\left(\;{\hat{y}}_{i}=y_{i}\;\right)$$

(3) $$\mathrm{Precision}\;=\frac{\mathrm{TP}}{\mathrm{TP}+\mathrm{FP}}\;$$

(4) $$\mathrm{Recall}\;=\frac{\mathrm{TP}}{\mathrm{TP}+\mathrm{FN}}\;$$

(5) $$F1\;\mathrm{Score}\;=\frac{2\cdot \mathrm{Precision}\cdot \mathrm{Recall}}{\mathrm{Precision}+\mathrm{Recall}}\;$$

where *TP* is the number of true positives, *FP* is the number of false positives, *FN* is the number of false negatives, 1(⋅) is the indicator function that returns 1 if the condition is true, and 0 otherwise. Hence, the proposed compact CNN architecture effectively extracted informative features from the acoustic sensor data to classify signals into 7 categories with over 96% accuracy across key metrics, demonstrating the feasibility of the sensors for speech recognition and their great potential for human‐computer interaction

## 3 Conclusion

We have demonstrated a sacrificial template‐full solution method for producing a blended thin film, which gained a rough surface and enables the application of a sandwich‐structured resistive pressure sensor. Transient absorption measurements and DFT analysis were applied to reveal the the advantages of employing a dopant in a sidechain‐structured CP thin film for resistive pressure sensor applications. This sensor was employed for diverse motion analyses and a vocal recognition system. We anticipate that this comprehensive approach will pave the way for new directions in the development of pressure sensors, offering opportunities for further advancements in this field.

## 4 Experimental Section

### Materials

Oligo ethylene glycol substituted polythiophene (Pg_3_2T‐TT), Trichloromethane, Acetonitrile, Propanone, tert‐Butyl phenyl carbonate (tBPC), 2,3,5,6‐Tetrafluoro‐7,7,8,8‐tetracyanoquinodimethane (F4TCNQ), Ferric chloride (FeCl_3_) were purchased from Sigma‐Aldrich. Indium‐tin oxide (ITO)‐coated poly(ethylene terephthalate) (PET) sheets (125µm‐thick)were bought from Huanan Xiangcheng Co., Ltd.

### Preparation of the Pressure Sensors

PF Sensor with Sacrificial Template: An amount of Pg_3_2T‐TT was weighed and dissolved in CHCl_3_ to configure a 5 mg mL^−1^ solution of Pg_3_2T‐TT, which was magnetically stirred at 50 °C for 4 h. F4TCNQ was weighed and dissolved in a solvent of CHCl_3_ and acetonitrile in a volume ratio of 9:1 to configure a 1 mg mL^−1^ solution of F4TCNQ. F4TCNQ solution was added to the Pg_3_2T‐TT solution and stirred until completely dissolved, and the volume ratio of F4TCNQ solution to Pg_3_2T‐TT solution was 10:11. The doped Pg_3_2T‐TT solution was mixed well with the tBPC solution and configured into sensitive layer solutions with different mixing ratios. into different volume ratios of the sensitive layer solution. The ITO/PET substrates were separately ultrasonic cleaned in acetone, deionized water and isopropyl alcohol for 15 min. The substrates were exposed to UV light (UV‐Ozone Cleaner) for 20 min. Take 25 µL of the prepared sensitive layer solution and drop it on the middle position of ITO/PET substrate, the area of ITO/PET is 20mm×10 mm, and the area covered by the solution is 10mm×10mm. after the film is formed, put the film into a glove box and heat it up at 78 °C for 30 min, and then take it out when it cools down naturally, and encapsulate it together with another ITO/PET substrate with the same size, and the sensor is ready.

### PF Sensors Without Sacrificial Templates

1mg mL^−1^ F4TCNQ solution and 5mg mL^−1^ Pg_3_2T‐TT solution were prepared as in the above preparation process and mixed until transparent. Take 25 µL of the mixed solution and drop‐cast it onto the ITO/PET substrate, and wait for the film to form naturally, then the preparation is completed.

### Characterizations and Measurements

The surface topography of the sensing layer was characterized using an optical microscope (WYJ‐910). The microscopic morphology of the surface and cross‐section of the sensing layer using SEM (5000X+UltimMax 40e). The surface roughness of the sensor was tested using a laser confocal microscope (KEYENCE VK‐X1000), and the 3D surface profile, 2D height map, and topographic data of the pressure sensor were obtained. The characterization data were used for 3D modeling and simulation of the sensor.

Transient absorption experimental set‐up. The light source for transient absorption spectra was an 800‐nm Ti: sapphire amplified pulsed laser which delivered 7 W pulses with a pulse width of 35 fs at a 10‐kHz repetition rate. The output light is split into two parts: one beam is used to generate a 500 nm pump pulse through an optical parametric amplifier, and the other beam is used to generate supercontinuum white light in a 3 mm thick sapphire window as probe pulse. The pump pulse was sent through an electronically controlled mechanical delay line (DL225) to adjust the optical path length, thereby controlling the time delay between the pump and probe pulses reaching the sample. The sample is irradiated by pump and probe pulses with pulse energies of 700 and 7.5 nJ, respectively. The spatial angle between the two beams is approximately 15°, and the polarization angle between the two beams is set to the magic angle to avoid noise caused by anisotropy. The probe pulse, after passing through the sample, is lead into a spectrometer and detected by a charge coupled device (CCD) camera. Transient absorption spectroscopy measurements with the doped samples were performed under both atmospheric and pressurized conditions and the undoped samples as the control group. The pressure for the pressurized group was applied by tightly pressing a glass substrate on the sample with a home‐made apparatus.

### The Sensing Performance Test of the Sensor

The transducer performance testing system consisted of a universal tensile tester (ZQ‐990B) and a source meter (Keithley 2450), which was connected and controlled by a computer. Specifically, the electrodes of the pressure transducer were connected to the source meter with a silver wire and a driving voltage of 1 V was supplied to the transducer through the source meter. The sensor was then squeezed by a press to different pressures, the pressure values are given by the press and the electrical output of the sensor is recorded by the computer. All human experiments were performed with approval from the Shanghai University Experimental Ethics Committee (PJ2024‐65, Certificate No. SYXK 2024‐0017). Written consent was acquired from the volunteer of the research.

## Conflict of Interest

The authors declare no conflict of interest.

## Supporting information



Supporting Information

Supplemental Movie 1

## Data Availability

The data that support the findings of this study are available in the supplementary material of this article.
