# Supplementary material for: Pressure Induced Molecular‐Arrangement and Charge‐Density Perturbance in Doped Polymer for Intelligent Motion and Vocal Recognitions
Source: Adv Mater. 2025 Apr 8;37(27):2500077. doi: 10.1002/adma.202500077 (PMC12243725; doi:10.1002/adma.202500077)
Supplement: Supplementary file 1 — Supporting Information [file ADMA-37-2500077-s002.docx]

**Supporting information**

**Pressure Induced Molecular-arrangement and Charge-density Perturbance in Doped Polymer for Intelligent Motion and Vocal Recognitions**

Huimin Lu ^a,b#^, Lei Zhang ^a,b#^, Jingyan Jiang ^c,#^, Jian Song ^a,b^*, Zhongchao Zhou ^a,b^, Wujian Wu ^c^, Ziqian Cheng ^d^, Tengfei Yan ^a^, Hong Hu ^a,b^, Tingting Zhao ^a^, Zhen Xu ^e^, Siyi Luo ^e^, Hui Li ^e,^*, Jianhua Zhang ^a,^* and Charles H. Lawrie^b,f,g,h,^*

^a^ School of Microelectronics, Shanghai University, Shanghai, 201800, China

^b^ Sino-Swiss Institute of Advanced Technology (SSIAT), Shanghai University, Shanghai, 201899, China

^c^ College of Big data and Internet, Shenzhen Technology University, Shenzhen, China

^d^ Graduate School of China Academy of Engineering Physics, Beijing, 100193, China

^e^ State Key Laboratory of High Performance Ceramics and Superfine Microstructures, Shanghai Institute of Ceramics Chinese Academy of Sciences, Shanghai, 200050, China

^f^ Biogipuzkoa Health Research Institute, San Sebastian, Spain

^g^ IKERBASQUE, Basque Foundation for Science, Bilbao, Spain

^h^ Radcliffe Department of Medicine, University of Oxford, Oxford, UK

*Corresponding Authors:

J. Song: [jsong@shu.edu.cn](mailto:jsong@shu.edu.cn)

H. Li: [lihui889@mail.sic.ac.cn](mailto:lihui889@mail.sic.ac.cn)

C. H. Lawrie: [charles.lawrie@bio-gipuzkoa.eus](mailto:charles.lawrie@bio-gipuzkoa.eus)

J. Zhang: jhzhang@oa.shu.edu.cn

# Huimin Lu, Lei Zhang and Jingyan Jiang contributed equally to this work.

**
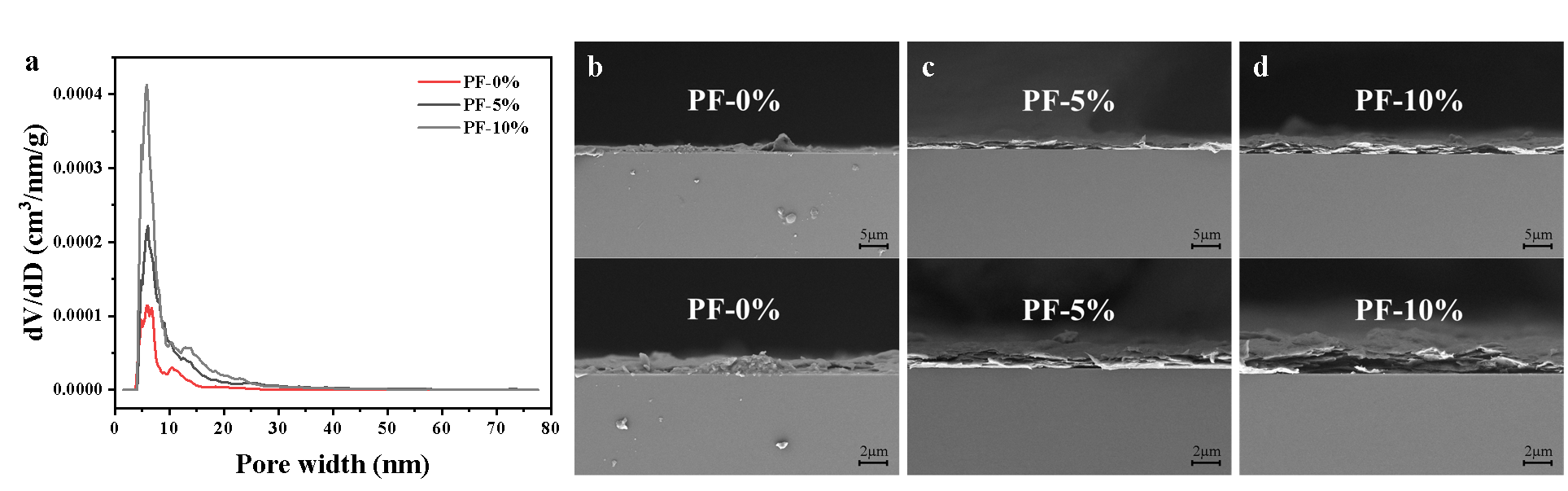
**

**Figure S1. a** Pore size distribution diagram. SEM images of **b** PF-0%, **c** PF-5%, **d** PF-10%.

**
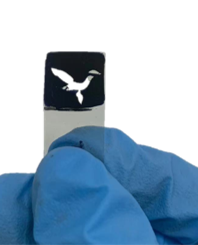
**

**Figure S2.** The morphology of PF-15% thin film.

We imported the height data measured by laser confocal into Matlab for analysis. The height data of each surface was shifted so that the valley with the largest absolute value was shifted to 0. The histograme function was used to initially determine the distribution of the surface heights. The mean surface roughness and root mean square roughness (R_q_) for PF-0%, PF-5% and PF-10% were 0.54, 0.99, 1.22 and 0.56, 1, 1.26, respectively. The lillietest function was used to determine if the surface conformed to a Gaussian surface. There are two return values h and p in the function, h is the hypothesis and p is the variance probability. The return values of the function for all three surfaces are 1 for h and 1e-3 for p. Therefore, the surface heights all conform to a Gaussian distribution.


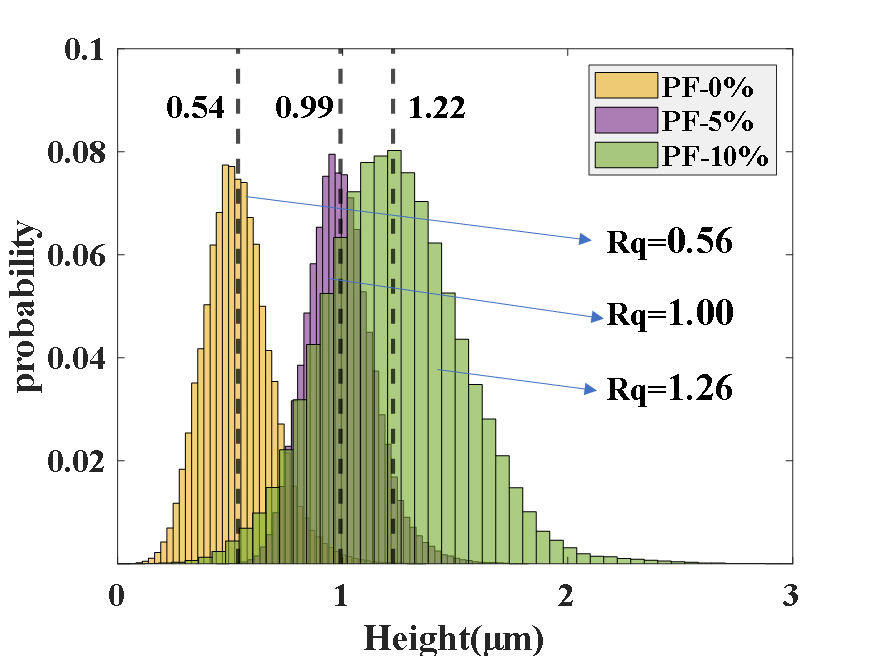


**Figure S3.** Surface Height Distribution of PF-0%, PF-5%, PF-10%.


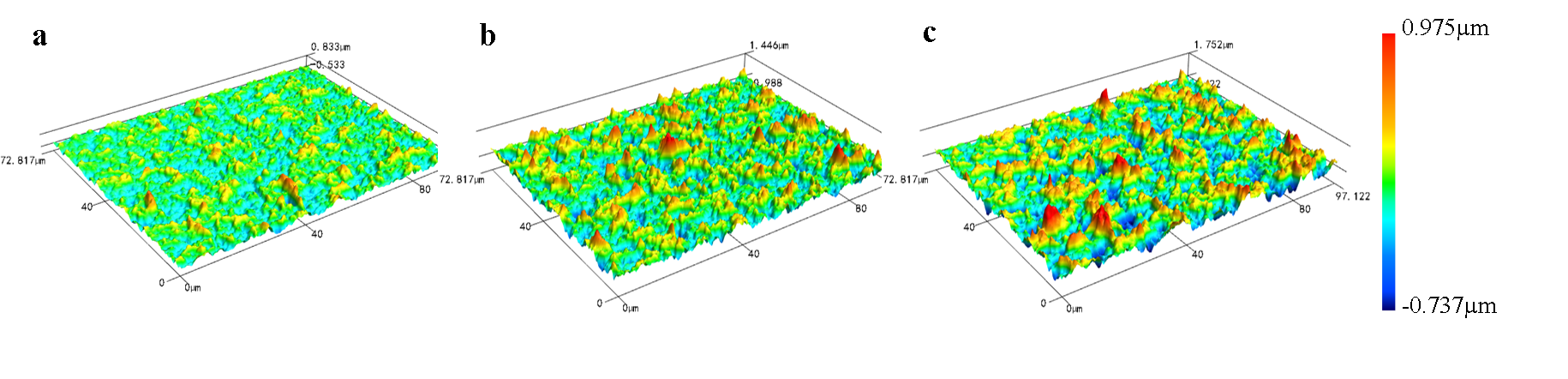


**Figure S4.** Three-dimensional surface morphology of **a** PF-0%, **b** PF-5%, **c** PF-10%.


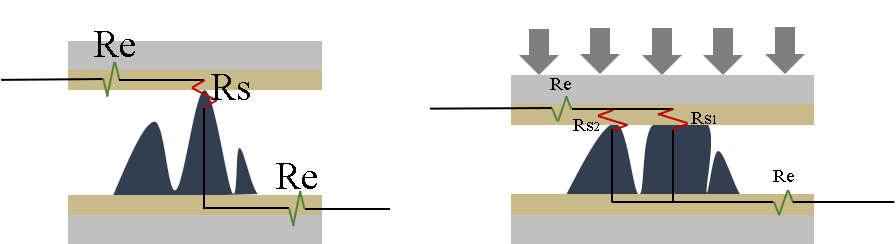


**Figure S5.** The equivalent resistance diagrams of the sensor


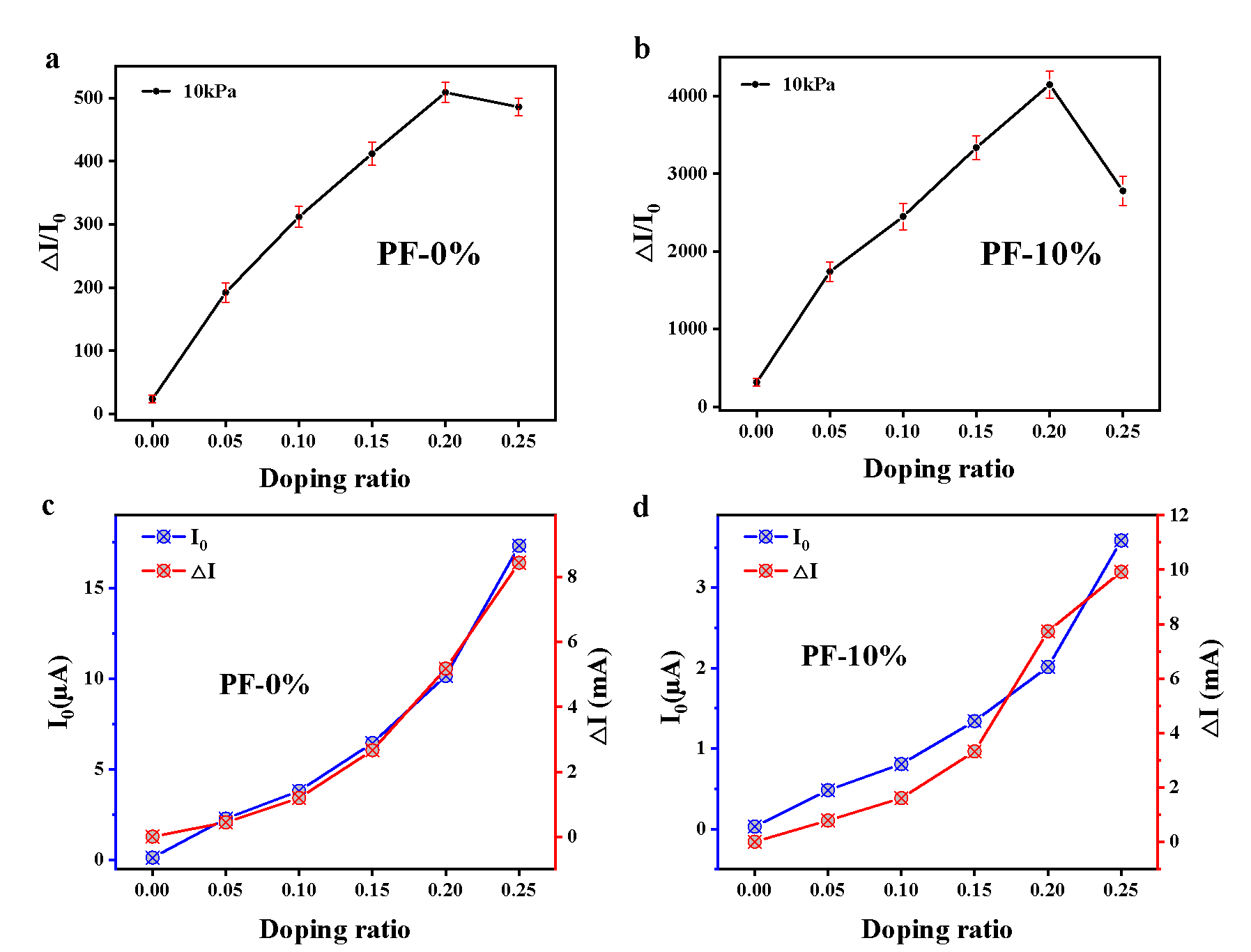


**Figure S6.** Response curves of **a** PF-0% and **b** PF-10% with different doping ratios at 10 kPa. I_0_ and ΔI at 10 kPa for **c** PF-0% and **d** PF-10% with different doping ratios.


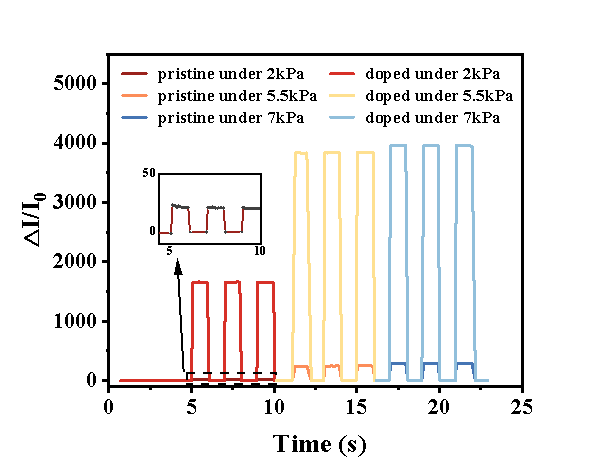


**Figure S7.** ΔI/I₀-T curve with and without dopant.


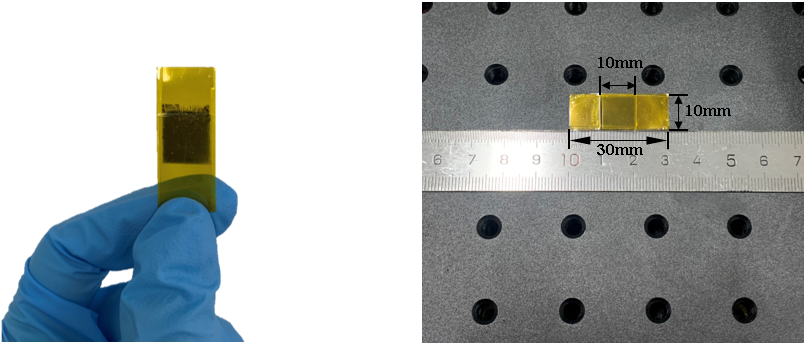


**Figure S8.** Physical drawing of PF-10% sensor.


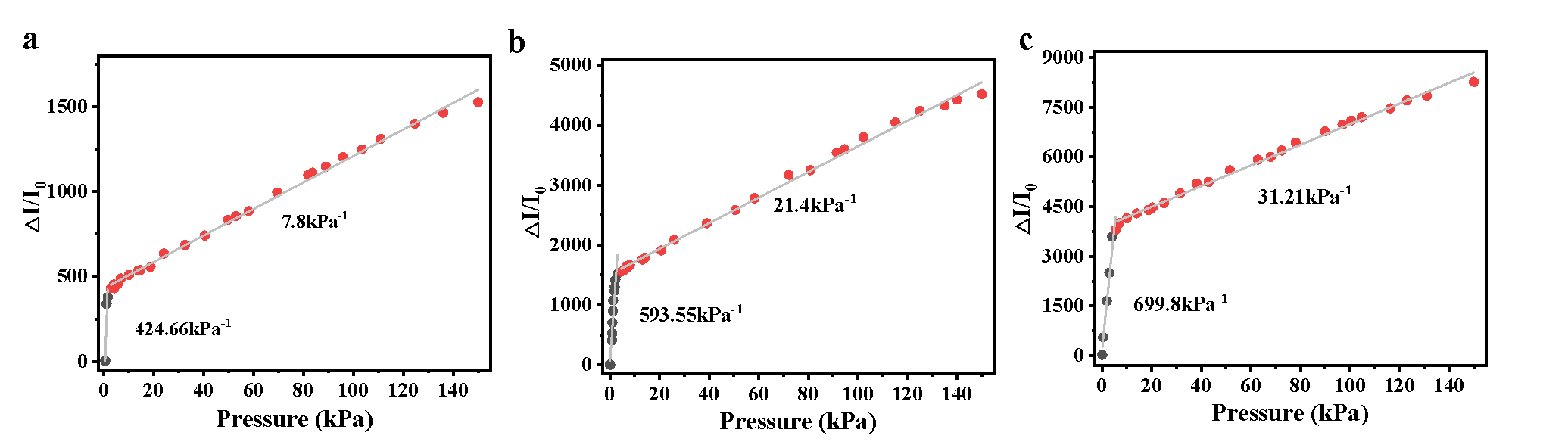


**Figure S9.** Sensitivities of PF-0%, PF-5%, and PF-10%.


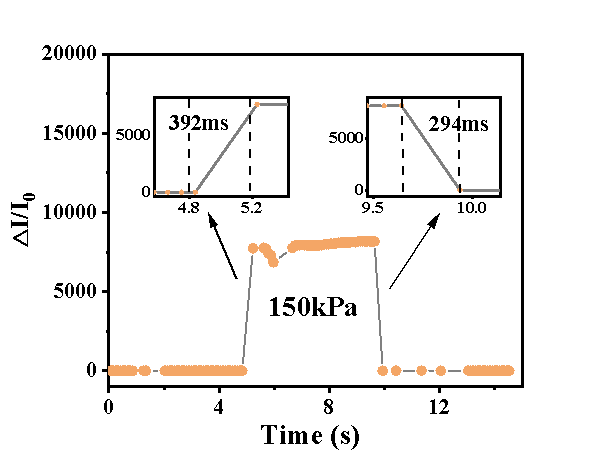


**Figure S10.** Response and recovery time of the sensor toward 150kPa.


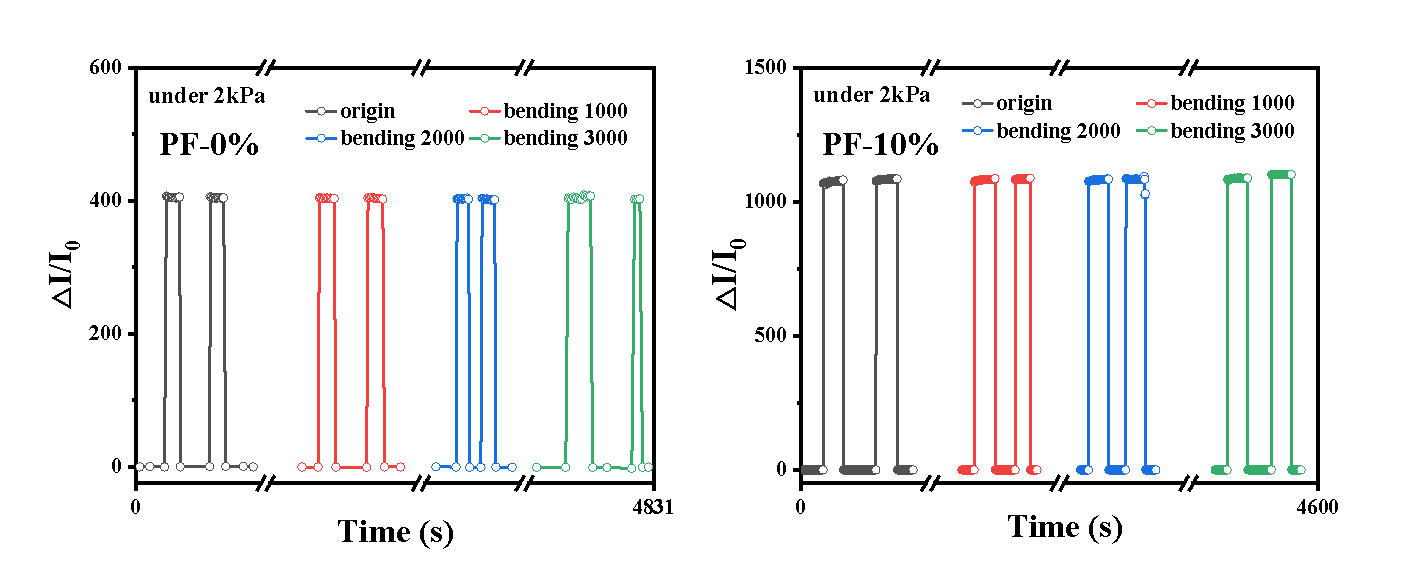


**Figure S11.** Cyclic bending test curves for PF-0% and PF-10%


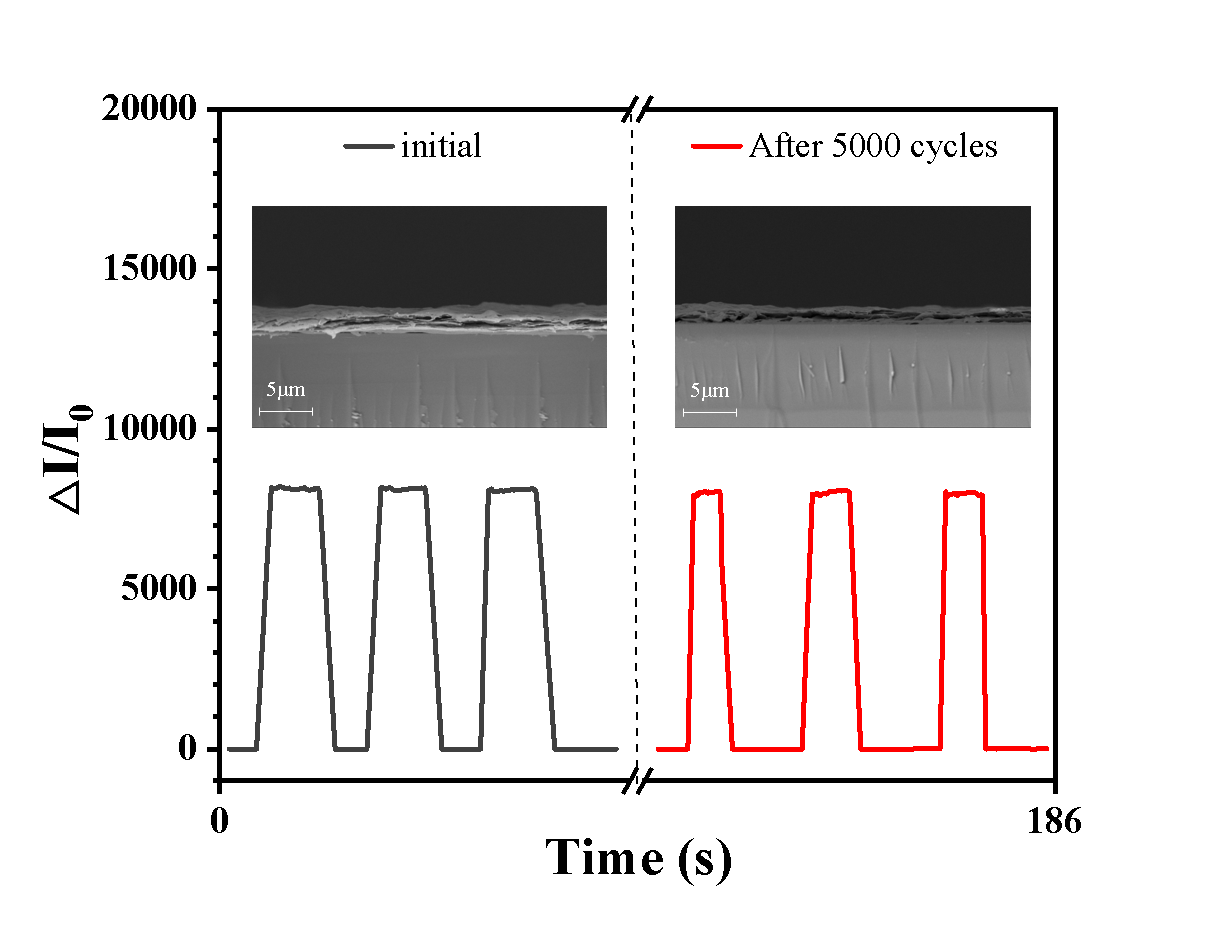


**Figure S12.** Response curves and SEM images before and after 5000 cycles under 150kPa pressure.


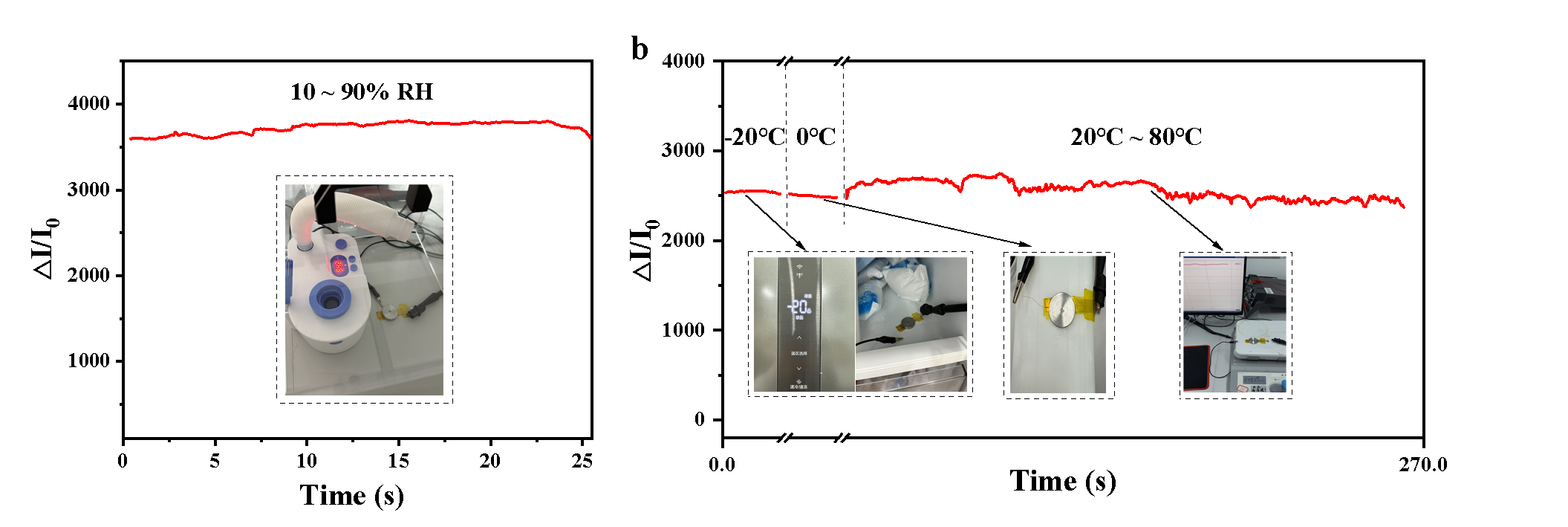


**Figure S13.** Sensing performance of **a** different humidity under 5 kPa, **b** different temperature under 2 kPa.


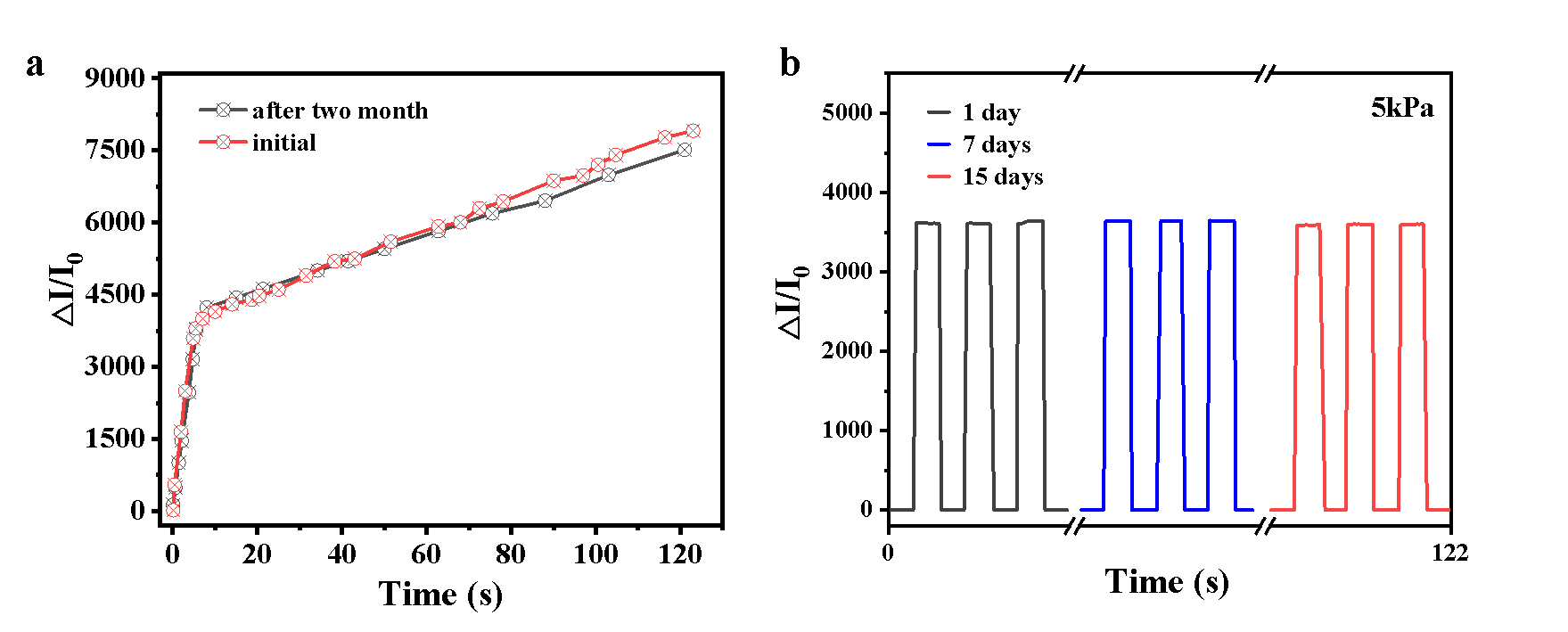


**Figure S14.** **a** Sensitivity curves before and after two months. **b** Weekly measurements of PF-10% under 5 kPa

**Supplementary Tables**

**Table S1.**

| **Materials** | **Sensitivity Ⅰ (kPa^−1^)** | **Linear range** | **Sensitivity Ⅱ (kPa^−1^)** | **Detection limit (Pa)** | **Response time (ms)** | **Years** | **Ref** |
| --- | --- | --- | --- | --- | --- | --- | --- |
| **PPy/PDMS** | **19.32** | **~0.5kPa** | **0.51** | **1** | **20/30** | **2018** | **（1）** |
| **MXene-FPDMS** | **14.2** | **~62Pa** | **3.66** | **3.4** | **90/74** | **2024** | **(2)** |
| **LBG/PVA/**  **CNTs-DR** | **20.5** | **~1 kPa** | **2.28** | **<100** | **356/355** | **2022** | **(3)** |
| **PEDOT:PSS/PDMS** | **21** | **~100Pa** | **0.016** | **<5** | **90** | **2020** | **(4)** |
| **MXene/PEDOT:PSS CompositeAerogel** | **26.65** | **~2kPa** | **3.07** | **-** | **106/95** | **2022** | **(5)** |
| **AgNWs/GR/PANF** | **130.4** | **~1.5kPa** | **3.5** | **3.7** | **20/20** | **2020** | **(6)** |
| **PVDF/PDA/PPy** | **139.9** | **~2kPa** | **19.6** | **0.9** | **22/53** | **2020** | **(7)** |
| **PEDOT:PSS/PDMS** | **642.5** | **0~1kPa** | **5.2** | **7.14** | **0.2/11.2** | **2021** | **（8）** |
| **MXene/silver nanowires** | **1434.89** | **0~5kPa** | **150.66** | **1** | **70/81** | **2022** | **（9）** |
| **MXene/**  **Bacterial** | **51.14** | **0.82** | **2.62** | **-** | **99** | **2022** | **（10）** |
| **Pg_3_2T‐TT/F4TCNQ** | **699.8** | **~5.4kPa** | **33.9** | **0.7** | **19.5/18.8** | **-** | **This work** |

**Supplementary Videos**

**Video S1.**

This sensor was attached on the mouse which was using for drawing on the computer. A sensing curve was obtained in real-time with mouse click, indicating the ability to respond dynamic changes in pressure as same as the sensor inside the mouse.

**Reference**

[1] C. Yang, L. Li, J. Zhao, J. Wang, J. Xie, Y. Cao, M. Xue, C. Lu, *ACS Appl. Mater. Interfaces* **2018**, *10*, 25811.

[2] W. Guo, Z. Ma, Z. Chen, H. Hua, D. Wang, M. Elhousseini Hilal, Y. Fu, P. Lu, J. Lu, Y. Zhang, D. Ho, B. L. Khoo, *Chem. Eng. J.* **2024**, *485*, 149659.

[3] Y. Huang, B. Liu, W. Zhang, G. Qu, S. Jin, X. Li, Z. Nie, H. Zhou, *Npj Flex. Electron.* **2022**, *6*, 92.

[4] J. J. Lee, S. Gandla, B. Lim, S. Kang, S. Kim, S. Lee, S. Kim, *NPG Asia Mater.* **2020**, *12*, 65.

[5] S. Zhang, T. Tu, T. Li, Y. Cai, Z. Wang, Y. Zhou, D. Wang, L. Fang, X. Ye, B. Liang, *ACS Appl. Mater. Interfaces* **2022**, *14*, 23877.

[6] X. Li, Y. J. Fan, H. Y. Li, J. W. Cao, Y. C. Xiao, Y. Wang, F. Liang, H. L. Wang, Y. Jiang, Z. L. Wang, G. Zhu, *ACS Nano* **2020**, *14*, 9605.

[7] H. Pan, G. Xie, W. Pang, S. Wang, Y. Wang, Z. Jiang, X. Du, H. Tai, *ACS Appl. Mater. Interfaces* **2020**, *12*, 38805.

[8] Z. Tan, H. Li, Y. Huang, X. Gong, J. Qi, J. Li, X. Chen, D. Ji, W. Lv, L. Li, W. Hu, *Compos. Part Appl. Sci. Manuf.* **2021**, *143*, 106299.

[9] X. Zheng, S. Zhang, M. Zhou, H. Lu, S. Guo, Y. Zhang, C. Li, S. C. Tan, *Adv. Funct. Mater.* **2023**, *33*, 2214880.

[10] T. Su, N. Liu, D. Lei, L. Wang, Z. Ren, Q. Zhang, J. Su, Z. Zhang, Y. Gao, *ACS Nano* **2022**, *16*, 8461.
